# Oral impact on daily performance (OIDP) scale for use in Sri Lankan adolescents: a cross sectional modification and validation study

**DOI:** 10.1186/s12903-020-1006-z

**Published:** 2020-01-21

**Authors:** Uttara Amilani, Prasanna Jayasekara, Irosha R. Perera, Hannah E. Carter, Sameera Senanayake, Sanjeewa Kularatna

**Affiliations:** 1 Family Health Bureau, Ministry of Health, Nutrition & Indigenous Medicine, 231 De Saram Place, Colombo, Sri Lanka; 2 National Cancer Control Programme, Ministry of Health, Nutrition & Indigenous Medicine, Colombo, Sri Lanka; 3 National Dental Hospital (Teaching), Ministry of Health, Nutrition & Indigenous Medicine, Colombo, Sri Lanka; 4 Australian Centre for Health Services Innovation, School of Public Health and Social Work, Faculty of Health, Queensland University of Technology, Brisbane, Queensland Australia

**Keywords:** Oral health, Quality of life, Adolescence, Validation studies, Factor analysis

## Abstract

**Background:**

Oral Health Related Quality of Life (OHRQoL) measures play an important role in understanding subjective patient experiences in oral health care. The Oral Impact on Daily Performance (OIDP) scale is a validated OHRQoL tool that measures the impact and extent to which an individual’s daily activities may be compromised by their oral health. It is commonly used to facilitate oral health service planning. The aim of this study was to modify and validate a Sinhalese version of the OIDP for use in Sri Lankan adolescents.

**Methods:**

Stage I involved cultural adaptation of the tool through translation and modification. Stage II involved the exploring factor structure, validation and a reliability assessment. After translation and cultural adaptation, stage II was conducted among 220 secondary school students aged 15–19 in the Gampaha district, Sri Lanka. Participants completed the modified OIDP scale along with questions on self-reported perceived oral health problems and treatment need which were used to assesses the concurrent validity of the modified OIDP scale. Factorability was assessed by inspection of correlation matrix and Kaiser-Meyer-Olkin and Bartlett’s Test of Sphericity tests as a measure of sampling adequacy. An exploratory factor analysis was carried out using Principal Component Analysis method and factors were rotated using the oblimin method.

**Results:**

The Kaiser-Meyer-Olkin measure was 0.87 and Bartlett’s test of Sphericity was significant (*p* < 0.001) Cronbach’s alpha was calculated as 0.88, indicating a high level of internal consistency of the modified OIDP scale. The principal component analysis produced two factors with Eigen values ranging from 1.12 to 4.40, explaining 70.0% of total variance. Concurrent validity was satisfactory as the OIDP score increased when the adolescents’ perceived oral health decreased. The final modified OIDP consists of eight self-reported items which assesses the impact severity of eight daily performances over past three months. Participant scores ranged from 0 to 24 out of a worst possible score of 40, and nearly 48% of the responders reported at least one impact during past three months. The most prevalent oral health impact related to chewing and enjoying foods, reported by 36.8% of respondents.

**Conclusion:**

This study suggests that the modified OIDP scale has promising psychometric properties and is appropriate for use among adolescents in Sri Lanka. Further research is required to test the validity of this tool in other cohorts.

## Background

Recent reports have identified an increase in the global prevalence of dental caries in both children and adults [1]. Poor oral health may have a profound effect on general health and experience of pain, including problems with eating, chewing, smiling and communication. Additionally, discolored and damaged teeth have a major impact on people’s daily living and wellbeing [2]. Research on quality of life informs estimates of the burden of illness and serves as criteria in identifying priority groups for public health interventions. It may also be used to establish outcome measures for oral health promotion activities [3].

The Oral Impact on Daily Performance (OIDP) is one of the most commonly used oral health related quality of life instruments globally. It measures the impact and extent to which the ability to perform regular physical, psychological and social activities is compromised due to poor oral health [4]. It has been developed to be used in conjunction with normative measures to assess population dental needs in order to facilitate oral health service planning. The instrument presents a good fit for use in population surveys due to the relatively low response burden [5] and its alignment with the international classification of impairments, disabilities and handicaps (ICIDH) [6], which has been amended for dentistry [7].

The OIDP has not yet been dimensionally validated for a Sri Lankan population. There is a lack of evidence available on the dimensional validity of the scale and whether it should be interpreted as a unidimensional or a multidimensional construct during cross cultural validation [8, 9]. Further, while the OIDP scale has been widely used globally, most studies were carried in cohorts of adults or younger children, with relatively few studies in adolescents [5, 10]. The aim of this study was to (1) culturally adapt a Sinhalese version of the OIDP for use in Sri Lankan adolescents and explore its factor structure; and (2) assess the psychometric properties and validate this modified version in a cohort of Sri Lankan adolescents.

## Methods

The OIDP is a self-administered instrument that measures the effect of oral impacts on an individual’s ability to perform eight daily performances: eating and enjoying food; speaking and pronouncing clearly; cleaning teeth; sleeping and relaxing; smiling; laughing and showing teeth without embarrassment; maintaining usual emotional state without being irritable; carrying out major work or social roles; and, enjoying contact with people. The total impact of each performance is calculated by multiplying a frequency score with a severity score. Frequency scores are obtained using the criteria used for the description of both frequency (for people affected on a regular or periodic basis) and the duration (for people affected for a period/spell) Severity scores are obtained by asking respondents to rate each item, ranging from 0 to 5, as an indication of how much it impacted on their daily living. The total score is the sum of all the performance scores for an individual. Then sum is divided by the maximum possible score and multiplying by 100 to give a percentage score [11].

**Fig. 1:**
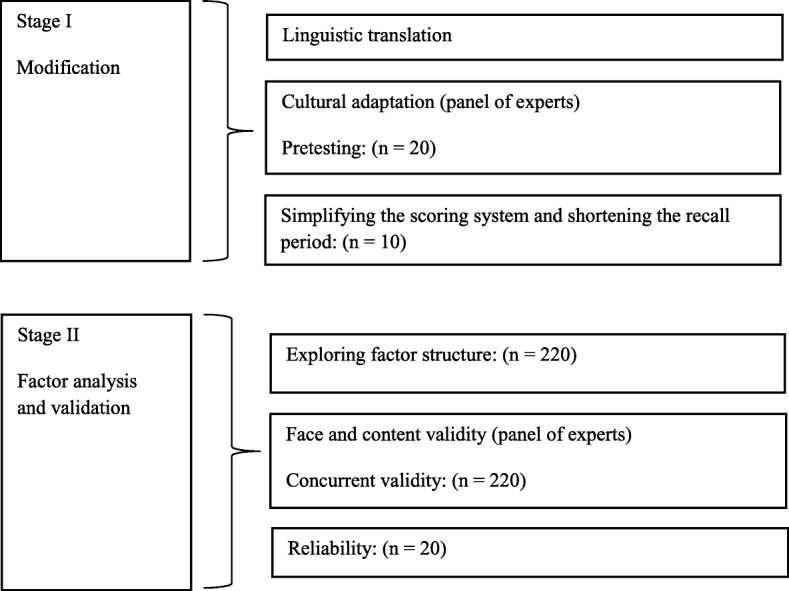
Schematic presentation of the modification and validation procedure of OIDP scale

The process of adapting the OIDP for Sri Lankan adolescents and evaluating of its psychometric properties involved two stages, summarized in Fig. 1. Both were conducted in Gampaha zone, Gampaha district, Sri Lanka. Stage I was carried out in the 1st quarter of 2015, followed by the stage II in the 2nd and 3rd quarters of 2015. Administrative clearance was obtained from the relevant education and health ministerial personnel and the study protocol was approved by Ethics Committee of Colombo Medical Faculty (Ref No EC 15–171).

### Stage I: modification for Sri Lankan adolescents

Stage I involved three main steps: linguistic translation; cultural adaptation and pretesting; and simplifying the scoring system and shortening the recall period. The process of cross-cultural adaptation including translation, adaptation and pretesting adopted the methods recommended by Guillemin and Beaton et al. [12, 13].

As the original version of the OIDP is in English, a Sinhalese translation was produced before any modifications were made. The original version of the OIDP was given to two translators whose first language was Sinhalese. Translation and back translation methods were applied and a third independent expert compared the back translated version with the original version and discrepancies were resolved with the consensus of the two translators [14].

The eight items of the modified OIDP were adapted for relevance to an adolescent population, while keeping the dimensions consistent with the original OIDP tool [11, 15] . A panel of experts including three specialists in community dentistry, two specialists in community medicine, two specialists in restorative dentistry, one specialist in orthodontics, one specialist in oral and maxillo-facial surgery and a sociologist were involved in this process. The experts were selected for their specialized knowledge, experience and unique perspectives on the content of the instrument [16, 17]. Public health expertise, clinical expertise, national representation and experience in research on the phenomenon of interest were used as the criteria when selecting content experts in to the panel of experts of the present study. Public health experts with experience in the designing and validation of measurement tools were prioritized. Adaptations are described in Table 1.

**Table 1:** Cultural adaptation and item modification of OIDP scale

No	Performances assessed in original tool	Item included in the modified tool
1	Eating and enjoying food	Impact on chewing and enjoying foods
2	Speaking and pronouncing clearly	Impact on talking and pronouncing clearly
3	Cleaning teeth	Impact on cleaning teeth
4	Sleeping and relaxing	Impact on good sleep without disturbances
5	Smiling, laughing and showing teeth without embarrassment	Impact on being able to smile without embarrassment
6	Maintaining usual emotional state without being irritable	Impacts on maintaining usual emotional state without being irritable
7	Carrying out major work or social role	Impact on school and household activities
8	Enjoying contact with people	Impact on enjoying time with friends

A draft of the modified OIDP scale was pretested by interviewing a convenient sample of 20 adolescents, aged 15–19 years. These participants were native Sinhalese speakers recruited from a secondary school in Gampaha Zone, Gampaha District. The Gampaha zone is located in the central part of the Gampaha district. It is an urbanized area with relatively high socio-economic indicators when compared to other zones in the District. The school was selected from the school list of the Gampaha Zone using a random number table. The interviewer recorded any difficulties that subjects had encountered, along with their comments. All records were reviewed by a study investigator and a discussion session with the interviewer. Six participants were followed up in order to clarify their comments. A series of re-interviews were conducted two weeks following the initial interviews in a subset of 10 participants in order to gain further insights into the scoring system and recall period.

The pre-testing process revealed that several adolescents gave a different set of responses in the re-interviews, unless the impacts were extremely low or extremely high. It was therefore determined by the panel of experts to limit the scoring system to a severity score only, as these responses were more consistent than those given for frequency. This is consistent with findings reported by the authors of the original instrument which suggest that, as the multiplication of both frequency and severity scores did not show any significant improvement over using the frequency or severity score alone, either the frequency or the severity score could be used alone for simplicity [11]. Modified OIDP scores were recorded on a six-point likert scale to reflect how severe the impact of each event was over the recall period, ranging from 0 (indicating no impact), to 5 (indicating a very severe impact). The total modified OIDP scores for individual domains were calculated as a simple sum of the response codes. Total modified OIDP scores could range from 0 to 40, where higher OIDP scores indicate poorer OHRQoL.

The pretesting further revealed that adolescents had poor memory of their oral health impacts over six months, as they gave different answers during the re-interview. The consensus of the panel of experts was therefore to shorten the recall period to three months. This is consistent with previous studies in children conducted in Brazil, France and India that also used the OIDP tool with a three months recall period [18–20], as well as a study that modified the OIDP scale for children in Thailand, without impacting on the validity of the tool [10] .

The final modified OIDP consists of 8 self-rated items which ask participants to assess the impact severity of eight daily performances over the past three months. A full list of modifications are presented in Additional file 1. The final modified OIDP is included in Additional file 2.

### Stage II

Stage II involved in exploring the factor structure and assessing the validity of the modified OIDP scale.

### Exploring factor structure

The factor analysis and the psychometric properties of the modified OIDP scale were assessed in a sample of 15–19 year school children from a secondary school in Gampaha zone, Dompe Medical officer of health area. Two classes were randomly selected from each grade (Grade 10 to Grade 13) to ensure the minimum sample size was met. The recommended minimum participant-to-item ratio in exploratory factor analysis is 5:1. A widely acceptable rule of thumb is 10:1 [21, 22]. We adopted a conservative 20:1 participant-to-item ratio to derive a minimum sample size of 160. A total of 220 participants from eight classes were recruited for the data collection. Data collection commenced by providing participants with the modified OIDP scale to be completed at the school premises during their class time as a self-administered instrument. All quantitative analyses were performed using the Statistical Package for Social Sciences (SPSS) version 23 by the study investigator. Socio-demographic data of the participants were described in frequency tables as numbers and percentages. No missing data were reported.

Using the approach described in Tabachnick and Fidell (2007), inspection of correlation matrix was performed to assess factorability [23]. Prior to proceeding further with factor extraction, Kaiser-Meyer-Olkin (KMO) a measure of Sampling Adequacy and Bartlett’s Test of Sphericity tests were performed. Williams (2010) has suggested that the KMO index should be at least 0.50 and Bartlett’s test of Sphericity should be significant (*p* < 0.05) to be considered suitable for factor analysis [24, 25].

Factor extraction is generally applied to reduce a large number of items into common groups or factors [14]. After assessing the factorability of the scale, the factor analysis of the eight items of the modified OIDP scale was conducted using Principal Component Analysis (PCA) and Principal Axis Factoring (PAF), the two most commonly used factoring procedures in published literature [23, 24, 26]. Simultaneous use of multiple decision rules, namely Kaiser’s criteria, Scree test and cumulative percent of variance extracted were recommended and considered [27]. Once the number of factors or components was decided, we adopted PCA with oblimin rotation which demonstrated a clearer and more interpretable structure relative to others methods. We adapted the PCA with oblimin rotation in order to allow factors to correlate, which is a low-risk, high benefit choice when compared to the orthogonal rotations [21]. Tabachnick and Fidell (2007) suggested that factor loading of 0.3 was a good rule of thumb for the minimum factor loading of an item [23]. A factor with a fewer than three items is generally weak and unstable; five or more strongly loading items (0.5) are desirable and indicate a solid factor [28]. Tabachnick and Fidell (2007) further advised that decisions about number of factors and appropriate rotational method should ultimately be based on realistic criteria, over an arbitrary rule of thumb [29]. These criteria were utilized during the selection of factors and relevant items for the modified OIDP scale.

### Validation

Psychometric analysis of the Sinhalese version of the modified OIDP involved the assessment of face, content and concurrent validity, as well as internal and test retest reliability assessment. The psychometric properties were assessed among the same sample that participated in the factor analysis. During that process, in addition to the modified OIDP scale, a questionnaire relating to perceived oral treatment need and perceived oral health problems were given to the participants.

Internal reliability was measured by using standardized Cronbach’s alpha coefficient, inter-item correlations and corrected item correlations [30]. It has been reported that Cronbach’s alpha coefficient should be at least 0.7 for early stage of research, 0.8 for basic research and 0.9 for clinical instruments and correlations need to be in moderate range, between 0.2 to 0.8 [14, 31].

In order to assess the test retest reliability, which provides an estimate of the degree to which the results are reproducible [32], a randomly selected subgroup of 20 participants from Stage II were given the modified OIDP scale to recomplete two weeks after their initial response. The total score of the two sets of data were compared to assess the correlation. As the modified OIDP scale presents continuous data which were not normally distributed, the non-parametric spearman rho test was used to calculate the total scores of the sub scales and for the total scale.

Since a gold standard measure cannot be identified to assess oral health related quality of life, criterion validity could not be achieved. Hence, face and content validity were assessed by ascertaining opinions from a second panel of experts [33]. The panel included three consultants in community dentistry, two consultants in community medicine, two consultants in restorative dentistry, one consultant in orthodontics and a sociologist. The panel members were selected based on the previous criteria used in selecting experts for the cross-cultural adaptation (stage I study). Each item in the instrument was checked for its relevance and appropriateness in the local context.

Concurrent validity was assessed by testing the modified OIDP scale against two subjective perceptions [10]; by assessing the self-reported perceived oral treatment need and perceived oral health problems. Due to the skewed nature of the modified OIDP scores, the non-parametric Kruksal-Wallis test was used to assess relationships between the modified OIDP and subjective perceptions.

## Results

Changes to item wordings were determined during the cultural adaptation process in Stage I and are outlined in Table 1. Specifically, the impact of carrying out major work or social roles was adapted to instead ask about the impact of oral health on school and household activities. The impact of smiling, laughing and showing teeth without embarrassment was adapted to instead ask about whether participants were able to smile without embarrassment.

A total of 220 participants were involved in the factor analysis study, with 100% of these students completing the questionnaire in full. Key characteristics of the sample are summarized in Table 2. There was a relatively equal distribution of genders with 50.9% male. The mean age was 16.2 (SD = 1.12) years.

**Table 2:** Demographic characteristics of the participants in factor analysis and validation study (*n* = 220)

Characteristics		N (%)
Age	15–16	71 (32.3)
	16–17	62 (28.2)
	17–18	43 (19.5)
	18–19	44 (20.0)
Sex	Male	112 (50.9)
	Female	108 (49.1)
Mother’s occupation category	Unemployed	187 (85.0)
	Primary	29 (13.2)
	Secondary	4 (1.8)
	Tertiary/ Senior	0 (0)
Father’s occupation category	Unemployed	9 (4.1)
	Primary	195 (88.6)
	Secondary	16 (7.3)
	Tertiary/ Senior	0 (0)

The mean completion time of the tool was approximately 5–8 min. Total scores ranged from 0 to 24 out of a worst possible score of 40. Approximately 48% of participants reported at least one impact during past three months. The most prevalent oral health impact was related to chewing and enjoying foods, reported by 36.8% of participants. Difficulties with talking and pronouncing clearly was reported by 21.4% of participants. The activities least affected by oral health were cleaning teeth and quality of sleep (both reported as being impacted by 12.3% of participants). Future details of the impacts reported in this stage are included in Additional file 3: Table S1.

An assessment of factorability found that all correlation coefficients were > 0.30 with no item found to increase Cronbach’s alpha when deleted. The KMO measure of sampling adequacy was 0.87 and Bartlett’s test of Sphericity was significant (*p* < 0.001), indicating that the data are suitable for factor analysis. Calculated Cronbach’s alpha for the study was 0.88, indicating good internal consistency reliability of the scale.

The exploratory factor analysis identified two factors using an Eigen value greater than one criterion. The factors were described as ‘social and psychological’ and ‘functional’ which is consistent with previous factor analyses of the OIDP [8, 9]. These two factors were able to explain 69.0% of the total variance. Item 5 was the only item loaded to two domains with factor coefficient more than 0.3. While the factor loading for this item was higher under the functional factor, the nature of the item determined that it would best fit under the ‘social and psychological’ factor; this is also consistent with previous studies [8, 9]. After the rotation, modified OIDP scale was prepared with items which scored more than 0.3 as factor loadings under a given factor (Table 3).

**Table 3:** Factor analysis of modified OIDP 8 items (*n* = 220)

	Modified OIDP items	Factor^a^	
Q	Question	Factor 1	Factor 2
Q1	Impact on chewing and enjoying foods	**0.545**	
Q2	Impact on talking and pronouncing clearly	**0.791**	
Q3	Impact on cleaning teeth	**0.960**	
Q4	Impact on good sleep without disturbances		**0.672**
Q5	Impact being able to smile without embarrassment	0.462	0.391
Q6	Impacts on maintaining usual emotional state without being irritable		**0.864**
Q7	Impact on school and household activities		**0.837**
Q8	Impact on enjoying time with friends		**0.910**

^a^ Results from oblimin rotation with Kaiser Normalization: Bold types indicates loading > 0.5

Factor 1: Functional, Factor 2: Social and Psychological

The inter item correlation coefficients among the 8 items of modified OIDP ranged from 0.18 (for the relationship between enjoying time with friends and teeth cleaning) to 0.72 (for the relationship between school and household activities and maintaining emotional status). The standard Cronbach’s alpha coefficient was 0.88. No correlation was negative indicating homogeneity among the items (Table 4).

**Table 4:** Reliability analysis: Inter-item correlation for the 8 items of the modified OIDP (*n* = 220)

Performance Scores	Chewing	Talking	Cleaning	Sleeping	Smiling	Emotion	Activities	Enjoying
Chewing	1.0							
Talking	0.50	1.0						
Cleaning	0.43	0.62	1.0					
Sleeping	0.51	0.54	0.39	1.0				
Smiling	0.33	0.62	0.39	0.52	1.0			
Emotion	0.42	0.43	0.24	0.60	0.48	1.0		
Activities	0.44	0.50	0.36	0.65	0.52	0.72	1.0	
Enjoying	0.36	0.40	0.18	0.59	0.41	0.58	0.67	1.0

The corrected item total correlations coefficients were between 0.48 to 0.75 and Cronbach’s alpha coefficient did not increase when any of the items were deleted (Table 5). These measures indicate the existence of important and significant relationships between the variables of the scale.

**Table 5:** Reliability Analysis: Corrected item- total correlations (*n* = 220)

Items	Corrected item-total correlations	Cronbach’s alpha if item deleted
Impact on chewing and enjoying foods	0.57	0.86
Impact on talking and pronouncing clearly	0.72	0.84
Impact on cleaning teeth	0.48	0.87
Impact on good sleep without disturbances	0.73	0.83
Impact on being able to smile without embarrassment	0.61	0.85
Impact on maintaining usual emotional state without being irritable	0.65	0.84
Impact on school and household activities	0.75	0.84
Impact on enjoying time with friends	0.59	0.85

A comparison of the correlations between test-retest scores in a sample of 20 participants two weeks apart was used to determine the stability of the modified OIDP. Spearman rho was calculated for each item’s scale and for the total scale. All correlations were positively associated in test and retest conditions. Spearman rho scores were 0.79 for the social and psychological factor, 0.76 for the functional factor, and 0.75 for the total scale. These relatively strong correlations indicate a high level of stability of the modified scale (Table 4).

Concurrent validity was assessed by testing modified OIDP scale against self-reported perceived oral treatment need and perceived oral health problems (Table 6). The relationships were significant (*p* < 0.05) indicating that the instrument could adequately discriminate between adolescents who had did not have perceived dental treatment needs and adolescents who had different perceptions of overall health problems.

**Table 6:** Concurrent validity test for the modified OIDP scores between different categories of related outcome variables (*n* = 220)

Variable	N	Mean	(SD)	*P* value^*^
Perceived oral treatment need				
Yes	82	2.60	3.85	0.003
No	103	1.56	4.00	
Don’t know	35	2.08	3.91	
Perceived oral health problems				
None	145	0.96	1.45	< 0.001
Little	56	3.27	5.71	
Moderate	15	5.41	5.91	
Severe	4	10.96	2.45	
Very severe	0	–	–	

*Kruksal-Wallis test was performed

## Discussion

This study was the first attempt to culturally adapt and dimensionally validate a Sinhalese version of the OIDP. The results from this study suggest that the modified, Sinhalese version of the OIDP scale has good reliability and excellent validity among a sample of 15 to 19 year aged adolescents in Sri Lanka, indicating its applicability for adolescent populations of similar ages in Sri Lanka more generally.

While preserving the relevant concepts and the validity of the original OIDP index, cross-cultural adaptation was performed in order to facilitate the direct cross-national and cross-cultural comparisons of international researches. We didn’t experience any major challenges in cross-cultural adaptation and we were able to obtain a good balance between the emic and etic perspective of the underlying theory of the scale. This may be due to the simple nature of the OIDP tool.

The modifications of the scale were based on adolescent’s capability in relation to their intellectual and cognitive development and as well as their memory recall ability. It was evident that adolescents had trouble in recalling impacts over the past six months. This is consistent with previous studies that used three month recall periods when measuring oral health related quality of life among children [10, 18–20, 34]. The OIDP scale for adolescents was therefore modified to have eight self-reported items with a three month recall period.

During the assessment of factor structure, PCA was applied and more than 68% of variance was explained by two factors; ‘social & psychological’ and ‘functional’, in addition to that it maximizes all variance in the items [35]. Patrick (1993) suggested that Health Related Quality of life (HRQoL) is a multidimensional construct including social, psychological and functional dimensions [36]. Being a subset of HRQoL, it is assumed that OHRQoL is a multidimensional construct as well [8]. Taken together, these findings suggested that OIDP fits within the conceptual and theoretical frameworks of multidimensionality in OHRQoL measures. However, further work using confirmatory factor analysis is needed to determine whether OIDP subscale scores appear to have unique meaning and whether multiple scores are beneficial in enabling the interpretation of the respondents’ OHRQoL status, as well as whether the total OIDP score could be reported as a single construct capturing overall OHRQoL status.

The OIDP frequency scores showed item-to-scale correlations without negative values that are similar to those obtained in previous applications internationally, and no correlation was high enough for any item to be redundant [10, 19]. Internal consistency reliability in terms of Cronbach alpha of 0.88 indicates excellent psychometric properties compared with the recommended level 0.7 as standard [37]. Previous applications of the OIDP scale to various populations have yielded internal consistency values ranging from 0.5 to 0.9 [5, 19, 38, 39].

The psychometric properties of survey instruments are dependent on the language and cultural context in which they are used, especially in health. Quality of life measurement is an outcome measure of the overall health of an individual. It is dynamic and depends on the social environment [32]. Concurrent validity was tested between modified OIDP scores and perceived oral treatment need and perceived oral health problems and significant relationships were found. This is consistent with previous applications of the OIDP scale [10, 18, 19, 32, 39–42]. These results emphasize that perceptions of oral health and treatment need are strongly associated with oral health quality of life; the better the perceptions, the lower the prevalence of oral impacts [19, 32]. The use of a culturally specific tool to assess the oral health related quality of life among adolescents has been found to generate results which can be readily translated to relevant recommendations to improve the oral health of populations [5].

The eight impact prevalence rates ranged from 12 to 37% and has a relatively high floor effect. This indicates that the oral health impact on their daily living was moderate in this study population. This was lower than in other studies in similar age groups [5, 19, 32]. This could be explained by different levels of disease burden, socio-demographic and socio-economic factors internationally. The most prevalent impact of ‘chewing’ is consistent with the findings on other populations using OIDP [5, 19, 32].

As the study was confined to 15–19-year-old school going adolescent cohort who can read, understand and write in Sinhala language in Sri Lanka, the results cannot be generalized to a wider population including those who are not proficient in Sinhala language. Further, results cannot be generalized to other countries without cross cultural validation. Further work is necessary to determine the construct validity of the scale. This may include advanced psychometric validation using Rasch analysis to support the process of data transformation from ordinal to interval-like, to explore the dimensional structure and to exploration of the association between modified OIDP scores and the clinical indicators of oral health.

## Conclusion

The provision of oral health care in adolescents should address not just their clinical dental need, but also their socio dental need, taking into consideration their perceptions in terms of the quality of life impacts of oral conditions on their daily life. Based on our findings, it can be concluded that the modified OIDP scale for adolescents has excellent validity and good reliability, and can be used as a practical measure of oral health related quality of life in 15–19 year-old adolescents in Sri Lanka. It has sound theoretical framework and good psychometric properties. It is also short and relatively quick to administer. As with all health-related quality of life measures, further evidence of its performance in different populations are necessary.

## Supplementary information

**Additional file 1.** Modifications to the OIDP for Sri Lankan adolescents.

**Additional file 2.** Final structure of the modified OIPD.

**Additional file 3: Table S1.** Percentage distribution of the impact (percentage of students affected) and mean scores with standard deveaitions (SD) for the 8 items in modified OIDP (*n* = 220).

## Data Availability

The datasets used and/or analyses during the current study are available from the corresponding author on reasonable request.

## Abbreviations

HRQoL: Health Related Quality of Life
OHRQoL: Oral health Related Quality of Life
OIDP: Oral Impact on Daily Performance
