# Supplementary material for: Oral impact on daily performance (OIDP) scale for use in Sri Lankan adolescents: a cross sectional modification and validation study
Source: BMC Oral Health. 2020 Jan 21;20:16. doi: 10.1186/s12903-020-1006-z (PMC6975056; doi:10.1186/s12903-020-1006-z)
Supplement: Supplementary file 1 — Additional file 1. Modifications to the OIDP for Sri Lankan adolescents. [file 12903_2020_1006_MOESM1_ESM.docx]

**Additional file 1**

Modifications to the OIDP for Sri Lankan adolescents

1. **Performance adaptation**

| **No** | **Performances assessed in original tool** | **Modifications made** |
| --- | --- | --- |
| 1 | Eating and enjoying food | No modification |
| 2 | Speaking and pronouncing clearly | Reworded “speaking” to “talking” |
| 3 | Cleaning teeth | No modification |
| 4 | Sleeping and relaxing | Changed to “good sleep without disturbances” |
| 5 | Smiling, laughing and showing teeth without embarrassment | Changed to “being able to smile without embarrassment” |
| 6 | Maintaining usual emotional state without being irritable | No modification |
| 7 | Carrying out major work or social role | Changed to “carrying out school and household activities” |
| 8 | Enjoying contact with people | Changed to “enjoying time with friends” |

1. **Scoring system**

| **Scoring system in the original tool** | **Scoring system in the modified OIDP** |
| --- | --- |
| *Frequency score calculation* | *Frequency score calculation* |
| Calculated by capturing both frequency (for the people affected on regular or periodic basis) and the duration (for people affected for a spell period). Score ranging from ‘0’ to ‘5’ | Removed |
| *Severity score calculation* | *Severity score calculation* |
| Likert type scale ranging from ‘0’ to ‘5’ where ‘5’represent ‘very severe’ and ‘0’ represent ‘none’ | No change |
| *Time frame* | *Time frame* |
| Past 6 months | Changed to past 3 months |
| *Total score calculation* | *Total score calculation* |
| The score representing the total impact on each performance was calculated by multiplying the frequency with the severity score. The total score is the sum of all the performance scores for an individual. | The score representing the total impact on each performance was calculated by summing up of all the severity scores for an individual. |
