# Supplementary material for: Oral impact on daily performance (OIDP) scale for use in Sri Lankan adolescents: a cross sectional modification and validation study
Source: BMC Oral Health. 2020 Jan 21;20:16. doi: 10.1186/s12903-020-1006-z (PMC6975056; doi:10.1186/s12903-020-1006-z)
Supplement: Supplementary file 2 — Additional file 2. Final structure of the modified OIPD. [file 12903_2020_1006_MOESM2_ESM.docx]

**Additional file 2**

Final structure of the modified OIPD

| **Item No** | **Factor No** | **Item description (impact during past three months)** | **Likert Scale response (severity score)** | | | | | |
| --- | --- | --- | --- | --- | --- | --- | --- | --- |
| 1 | 1 | Impact on chewing and enjoying foods | 0 | 1 | 2 | 3 | 4 | 5 |
| 2 | 1 | Impact on talking and pronouncing clearly | 0 | 1 | 2 | 3 | 4 | 5 |
| 3 | 1 | Impact on cleaning teeth | 0 | 1 | 2 | 3 | 4 | 5 |
| 4 | 2 | Impact on good sleep without disturbances | 0 | 1 | 2 | 3 | 4 | 5 |
| 5 | 2 | Impact being able to smile without embarrassment | 0 | 1 | 2 | 3 | 4 | 5 |
| 6 | 2 | Impacts on maintaining usual emotional state without being irritable | 0 | 1 | 2 | 3 | 4 | 5 |
| 7 | 2 | Impact on school and household activities | 0 | 1 | 2 | 3 | 4 | 5 |
| 8 | 2 | Impact on enjoying time with friends | 0 | 1 | 2 | 3 | 4 | 5 |

Scale response: (0) no impact, (1) very little impact, (2) little impact, (3) moderate impact, (4) severe impact, (5) very severe impact

Factor 1: Functional

Factor 2: Social and psychological
