# Supplementary material for: Oral impact on daily performance (OIDP) scale for use in Sri Lankan adolescents: a cross sectional modification and validation study
Source: BMC Oral Health. 2020 Jan 21;20:16. doi: 10.1186/s12903-020-1006-z (PMC6975056; doi:10.1186/s12903-020-1006-z)
Supplement: Supplementary file 3 — Additional file 3: Table S1. Percentage distribution of the impact (percentage of students affected) and mean scores with standard deveaitions (SD) for the 8 items in modified OIDP (n = 220). [file 12903_2020_1006_MOESM3_ESM.docx]

**Additional file 3**

**Table** Percentage distribution of the impact (percentage of students affected) and mean scores with standard deveaitions (SD) for the 8 items in modified OIDP (n=220)

| Item | % affected (N) | Mean score; 0-5 (SD) |
| --- | --- | --- |
| Impact on chewing and enjoying foods | 36.8 (81) | 0.45 (0.80) |
| Impact on talking and pronouncing clearly | 21.4 (47) | 0.23 (0.57) |
| Impact on cleaning teeth | 12.3 (27) | 0.29 (0.89) |
| Impact on good sleep without disturbances | 12.3 (27) | 0.23 (0.72) |
| Impact on being able to smile without embarrassment | 12.7 (28) | 0.21 (0.61) |
| Impacts on maintaining usual emotional state without being irritable | 13.2 (29) | 0.23 (0.66) |
| Impact on school and household activities | 13.2 (29) | 0.19 (0.47) |
| Impact on enjoying with friends | 13.2 (29) | 0.22 (0.64) |
| Total score (experience at least one impact) | 47.3 (104) | 2.03 (3.91) |
